# Evaluation of the Use of Cancer Registry Data for Comparative Effectiveness Research

**DOI:** 10.1001/jamanetworkopen.2020.11985

**Published:** 2020-07-30

**Authors:** Abhishek Kumar, Zachary D. Guss, Patrick T. Courtney, Vinit Nalawade, Paige Sheridan, Reith R. Sarkar, Matthew P. Banegas, Brent S. Rose, Ronghui Xu, James D. Murphy

**Affiliations:** 1 School of Medicine, University of California, San Diego, La Jolla; 2 Department of Radiation Medicine and Applied Sciences, University of California, San Diego, La Jolla; 3 Department of Radiation Oncology, The Johns Hopkins University, Baltimore, Maryland; 4 Center for Health Research, Kaiser Permanente Northwest, Portland, Oregon; 5 Division of Biostatistics and Bioinformatics, Department of Family Medicine and Public Health, University of California, San Diego, La Jolla; 6 Department of Mathematics, University of California, San Diego, La Jolla

## Abstract

**Importance:**

Researchers often analyze cancer registry data to assess for differences in survival among cancer treatments. However, the retrospective, nonrandomized design of these analyses raises questions about study validity.

**Objective:**

To examine the extent to which comparative effectiveness analyses using observational cancer registry data produce results concordant with those of randomized clinical trials.

**Design, Setting, and Participants:**

In this comparative effectiveness study, a total of 141 randomized clinical trials referenced in the National Comprehensive Cancer Network Clinical Practice Guidelines for 8 common solid tumor types were identified. Data on participants within the National Cancer Database (NCDB) diagnosed between 2004 and 2014, matching the eligibility criteria of the randomized clinical trial, were obtained. The present study was conducted from August 1, 2017, to September 10, 2019. The trials included 85 118 patients, and the corresponding NCDB analyses included 1 344 536 patients. Three Cox proportional hazards regression models were used to determine hazard ratios (HRs) for overall survival, including univariable, multivariable, and propensity score–adjusted models. Multivariable and propensity score analyses controlled for potential confounders, including demographic, comorbidity, clinical, treatment, and tumor-related variables.

**Main Outcomes and Measures:**

The main outcome was concordance between the results of randomized clinical trials and observational cancer registry data. Hazard ratios with an NCDB analysis were considered concordant if the NDCB HR fell within the 95% CI of the randomized clinical trial HR. An NCDB analysis was considered concordant if both the NCDB and clinical trial *P* values for survival were nonsignificant (*P* ≥ .05) or if they were both significant (*P* < .05) with survival favoring the same treatment arm in the NCDB and in the randomized clinical trial.

**Results:**

Analyses using the NCDB-produced HRs for survival were concordant with those of 141 randomized clinical trials in 79 univariable analyses (56%), 98 multivariable analyses (70%), and 90 propensity score models (64%). The NCDB analyses produced *P* values concordant with randomized clinical trials in 58 univariable analyses (41%), 65 multivariable analyses (46%), and 63 propensity score models (45%). No clinical trial characteristics were associated with concordance between NCDB analyses and randomized clinical trials, including disease site, type of clinical intervention, or severity of cancer.

**Conclusions and Relevance:**

The findings of this study suggest that comparative effectiveness research using cancer registry data often produces survival outcomes discordant with those of randomized clinical trial data. These findings may help provide context for clinicians and policy makers interpreting observational comparative effectiveness research in oncology.

## Key Points

**Question:**

Are the findings of comparative effectiveness analyses using cancer registry data concordant with the data from randomized clinical trials?

**Findings:**

In this comparative effectiveness study replicating 141 randomized clinical trials in the National Cancer Database, concordant hazard ratios for overall survival were noted in 56% to 70% of the analyses and concordant *P* values were noted in 41% to 46% of the analyses. No particular clinical trial features appeared to be associated with concordant hazard ratios.

**Meaning:**

The findings of this study suggest that comparative effectiveness research using the National Cancer Database often produces results discordant with randomized clinical trials.

## Teaser

This comparative effectiveness study examines the concordance of data from an observational cancer registry with data from randomized clinical trials.

## Introduction

Randomized clinical trials in oncology represent the highest standard level of evidence from which we establish efficacy of different therapeutic approaches.^1^ Despite the importance of randomized clinical trials, this study design has limitations associated with cost, timeliness, and generalizability of results in a real-world oncology population.^2,3,4^ In addition, several clinical scenarios encountered within oncology lack data from randomized settings to support clinical decision-making. To help fill these evidence gaps, investigators will often rely on research using nonrandomized observational data.

Cancer registries within the field of oncology represent a resource for the study of questions about the comparative benefits of different cancer therapies. Cancer registries collect detailed data on incident cancer diagnoses across a substantial portion of the US population.^5,6,7^ These resources play a role in the study of cancer incidence, prevalence, disparities, and patterns of care across the US. However, researchers have increasingly used cancer registry data to evaluate the comparative efficacy of different cancer treatments. The accessibility, ease of use, large sample sizes, and real-world nature of cancer registry data attract comparative effectiveness research. However, one must also consider the potential limitations of using these data for comparative effectiveness research. These limitations include concerns about accuracy with data collection,^8^ rigor of analytic techniques,^9^ or the potential for selection bias.^10^ Together, these limitations could skew results and threaten the validity of comparative effectiveness research with cancer registry data.

Within the field of oncology, we lack a clear understanding of how the survival outcomes of comparative effectiveness research with cancer registry data compare with those of the standard randomized clinical trials. An improved understanding of this relationship may help clinicians, researchers, and others who consult medical literature to better assess the role of observational research in the comparative effectiveness landscape. The purpose of this study was to measure agreement (ie, concordance) in survival estimates between randomized clinical trials and comparative effectiveness research with cancer registry data. Furthermore, we sought to identify features of clinical research questions that may lead comparative effectiveness research with cancer registry data to align more closely with results of randomized clinical trials.

## Methods

This study identified randomized clinical trials from the literature and compared their survival outcomes with outcomes of patient cohorts systematically analyzed from the National Cancer Database (NCDB).^11^ Analysis of the data was performed from August 1, 2017, to September 10, 2019. We included common cancer sites, such as the central nervous system, head and neck, breast, lung, gastrointestinal tract (anal, esophageal, gastric, pancreatic, and colorectal cancers), genitourinary system (bladder, kidney, and prostate cancers), and gynecologic system (uterine and cervical cancers) as well as lymphoma. This study was deemed exempt from institutional review board approval by the University of California, San Diego, because of the use of deidentified data. This study followed the International Society for Pharmacoeconomics and Outcomes Research (ISPOR) reporting guideline for comparative effectiveness research.

### Data Sources

**Figure 1.**
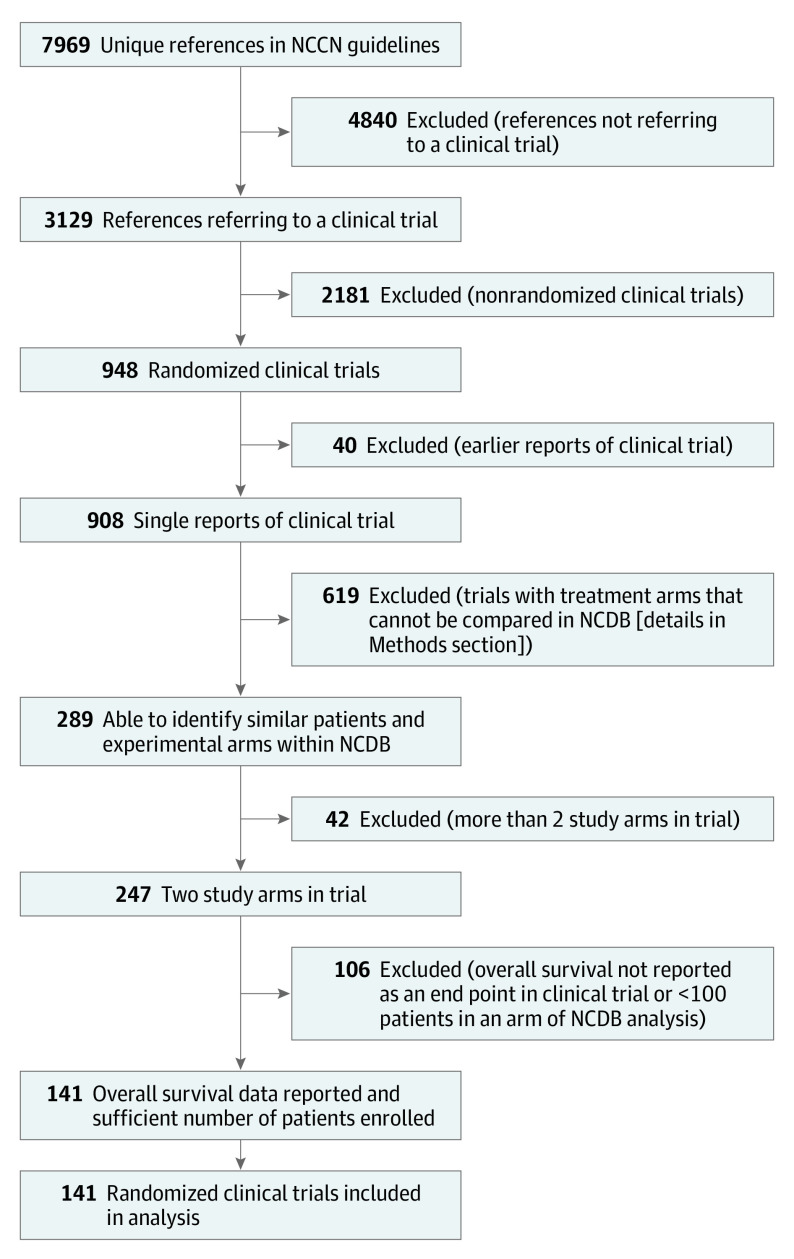
Identification of Randomized Clinical Trials NCCN indicates National Comprehensive Cancer Center Network; NCDB, National Cancer Database.

We systematically identified randomized clinical trials referenced in the National Comprehensive Cancer Network Clinical Practice Guidelines in Oncology (NCCN Guidelines) for each disease site.^12,13,14,15,16,17,18,19,20,21,22,23,24,25,26,27,28^ The NCCN Guidelines represent comprehensive tumor site–specific, evidence-based, consensus-driven guidelines that describe current treatment recommendations. The NCCN Guidelines also include a comprehensive written discussion describing in detail the clinical evidence supporting these recommendations. From the NCCN Guidelines we extracted 7969 referenced publications and reviewed these references to identify therapeutic randomized clinical trials involving surgery, systemic therapy, hormonal therapy, or radiotherapy. In scenarios in which a clinical trial’s results were reported in more than 1 publication (typically an original publication followed by an update) we used the most recent publication. Figure 1 depicts our clinical trial selection process, with additional exclusion criteria noted.

The NCDB represents a nationwide, facility-based cancer registry sponsored by a collaboration between the American College of Surgeon’s Commission on Cancer and the American Cancer Society.^11^ Trained tumor registrars at individual facilities record patient demographic, tumor, and treatment-related information on all incident cancers diagnosed at that facility according to standard protocols set forth by the American College of Surgeons.^29^ Specific information recorded includes TNM stage, tumor histologic characteristics, and grade, as well as details of treatment, including surgery (site, extent of surgery, and open vs laparoscopic) and radiotherapy (dose, number of fractions, and treatment modality). In addition, the NCDB records the use of chemotherapy, hormonal therapy, and immunotherapy, but does not report information on specific agents or duration of treatment. The Charlson comorbidity index score is measured the year before diagnosis.^30^ This study included patients within the NCDB diagnosed with cancer between 2004 and 2014.

### Eligibility

With each randomized clinical trial, we created a cohort of NCDB patients that matched the eligibility criteria of the trial whenever possible. We restricted each NCDB cohort to the disease site in question, histologic confirmation of cancer, TNM stage per trial eligibility, patient age 18 years or older, and no previous diagnosis of cancer. We further restricted each NCDB cohort to select patients receiving treatments delivered in each randomized clinical trial. For example, if a randomized clinical trial in breast cancer evaluated the effect of mastectomy alone compared with lumpectomy followed by radiotherapy, we would include only NCDB patients with a mastectomy alone or those who underwent a lumpectomy followed by radiotherapy. Owing to a lack of available data on specific systemic agents, we were unable to include randomized clinical trials comparing single-agent with multiagent systemic regimens or randomized clinical trials evaluating 2 different systemic agents. We included randomized clinical trials in which one arm contained a systemic agent and the other arm did not, with the limitation that the specific agent used was unknown. eTable 1 in the Supplement includes example descriptions of randomized clinical trial types included and excluded from this analysis.

 Randomized clinical trials often have eligibility criteria that include patient performance status or baseline laboratory values. Similar to other cancer registries, the NCDB does not contain performance status or laboratory data; therefore, these factors were not considered in our analysis. For trials involving radiotherapy, we restricted radiation doses to within 10 Gy of the trial-specified dose. If the trial specifically compared 2 different radiation doses, we restricted the NCDB cohort to the specified dose and number of fractions delivered in the trial. Within the NCDB cohorts, we defined chemotherapy delivered concurrently with radiotherapy if the start dates for both modalities occurred within 14 days of each other.

### Statistical Analysis

Of the randomized clinical trials included in this analysis, we recorded the hazard ratio (HR) for overall survival along with the 95% CI and associated *P* value. For trials that did not present an HR for overall survival in the article, we estimated the HRs from Kaplan-Meier plots.^31^ With each cohort of NCDB patients, we determined the HR for overall survival using 3 different analytic approaches: (1) unadjusted univariable Cox proportional hazards model, (2) multivariable Cox proportional hazards model, and (3) propensity score–adjusted analysis. All models measured survival from the date of diagnosis through death from any cause, censoring at the date of the last follow-up. Multivariable models included known confounding variables available in the NCDB that could potentially be factors in survival. At a minimum, each multivariable model included patient age, sex, race, geographic region, median household income, Charlson comorbidity index score, year of diagnosis, grade, site-specific histologic characteristics, and clinical or pathologic TNM stage. Individual multivariable models included additional factors related to specific disease sites, including *ER*, *PR*, and *ERBB2* for breast cancer, as well as prostate-specific antigen and Gleason score for prostate cancer. Patients with missing data on variables related to patient selection (eg, tumor site, stage, or treatment) were excluded from the analysis.

We derived propensity scores for each NCDB cohort from multivariable logistic regression models, using the same covariates noted above for the multivariable analyses. With each propensity score analysis, we used the inverse probability of the treatment-weighting approach and assessed balance between weighted covariates with mean standardized differences.

We assessed concordance between the randomized clinical trials and corresponding NCDB analyses using 3 different approaches. First, we assessed the association between HRs for overall survival between the randomized clinical trial and NCDB analysis with a Pearson correlation coefficient comparing log-transformed HRs, with a score of 0 indicating no correlation and 1 indicating perfect correlation. Second, we assessed concordance in HRs, with an NCDB analysis considered concordant if the NDCB HR fell within the 95% CI of the HR from the randomized clinical trial.^32^ Third, we considered concordance with respect to statistical significance, with an NCDB analysis considered concordant if both the NCDB and randomized clinical trial *P* values for survival were nonsignificant (*P* ≥ .05) or if they were both significant (*P* < .05), with survival favoring the same treatment arm in the NCDB and in the randomized clinical trial.

Univariable logistic regression was used to identify whether certain features of a randomized clinical trial would lead to higher levels of concordance in HRs between the randomized clinical trial and NCDB analysis. We evaluated the following features: domestic vs international setting, year of trial publication, tumor site, type of clinical trial question addressed, difference in mean age between NCDB treatment arms, aggressiveness of the cancer (assessed with the proxy of median survival of study population), and whether the randomized clinical trial primary end point was overall survival vs another primary end point. We also addressed the effect of trials with treatment arms of different durations to indirectly address immortal time bias.^33,34,35^ A study was defined as susceptible to immortal time bias if one treatment arm included lengthier treatment than the other treatment arm, such as with a randomized clinical trial comparing surgery alone with surgery followed by chemotherapy. Statistical analyses were performed using SAS, version 9.4 (SAS Institute Inc), with 2-sided *P* values < .05 determined using an unpaired, 2-tailed *t* test considered statistically significant.

## Results

One hundred forty-one randomized clinical trials met the inclusion criteria (Figure 1), with characteristics of the studies provided in Table 1 and a complete list of trial references provided in the eReferences in the Supplement. In general, most randomized clinical trials were published between 2004 and 2014 (92 [65%]) and took place outside North America (88 [62%]). The trials addressed an array of clinical questions (eg, addition of radiotherapy, 18 [13%] and addition of surgery, 6 [4%]), and nearly half evaluated the utility of adding systemic therapy to surgery or radiotherapy (67 [48%]). In total, the clinical trials included 85 118 patients, and the corresponding NCDB analyses included 1 344 536 patients. The median size of a clinical trial was smaller than the median size of an NCDB analysis (396 vs 6543 patients).

**Table 1.** Characteristics of Randomized Clinical Trials

Characteristic	No. (%)
Year of trial publication	
2003 or earlier	31 (22)
2004-2014	92 (65)
2015 or later	18 (13)
Geographic region of trial	
North America	43 (30)
Outside North America	88 (62)
Both	10 (7)
Anatomic site of tumors	
Breast	19 (13)
Central nervous system	8 (6)
Gastrointestinal	47 (33)
Genitourinary	18 (13)
Gynecologic	13 (9)
Head and neck	14 (10)
Lymphoma	6 (4)
Lung	16 (11)
Research question	
Addition of	
Systemic therapy	67 (48)
Radiotherapy	18 (13)
Radiotherapy and systemic therapy	11 (8)
Surgery	6 (4)
Radiation dose	11 (8)
Timing of treatment	5 (4)
Type of surgery	6 (4)
Type of systemic therapy	7 (5)
Other	10 (7)

**Figure 2.**
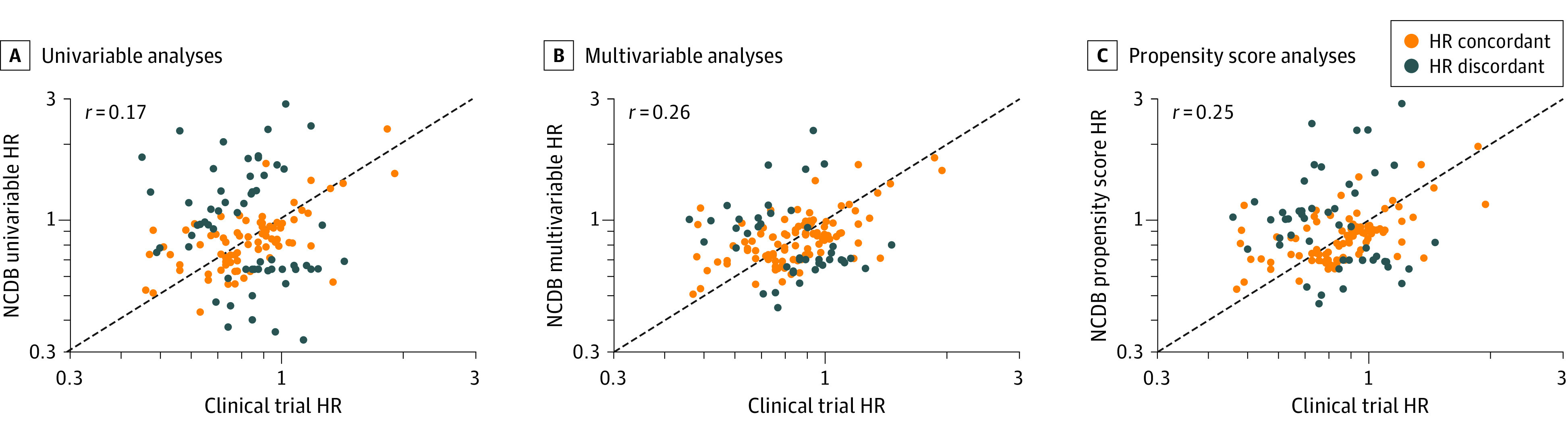
Comparison of Hazard Ratios (HRs) From Randomized Clinical Trials and Analyses With Data From the National Cancer Database (NCDB) Hazard ratios shown for univariable analyses (A), multivariable analyses (B), and propensity score analyses (C). Each point on the scatter plot represents the HR for overall survival from 1 of the 141 randomized clinical trials in this study and the corresponding analysis within the NCDB. Yellow dots represent NCDB analyses in which the NCDB HR falls within the 95% CI of the HR in the clinical trial (concordant), and blue dots represent HRs from the NCDB analyses that fall outside the 95% CI of the HR in the clinical trial (discordant). The gray dashed line shows where clinical trial HRs equal NCDB HRs. The intersection of the axes represents an HR equal to 1.

When considering all randomized clinical trials, we found a correlation between the HRs from the clinical trials and the HRs from the NCDB analyses for all 3 statistical analysis approaches (Figure 2). The correlation between the HR from the clinical trials and the NCDB was lowest with the unadjusted analysis (*r* = 0.17; 95% CI, 0.005-0.33; *P* = .02), followed by the propensity score analysis (*r* = 0.25; 95% CI, 0.09-0.40; *P* = .003), and the multivariable analysis (*r* = 0.26; 95% CI, 0.10-0.41; *P* = .003).

When evaluating concordance in HRs for overall survival, we found that 79 of 141 randomized clinical trials (56%) evaluated in the NCDB with a univariable analysis produced HRs concordant with those of randomized clinical trials. With multivariable analysis, 98 trials (70%) analyzed in the NCDB produced HRs concordant with those of randomized clinical trials. With propensity score analysis, 90 randomized clinical trials (64%) analyzed in the NCDB produced HRs concordant with those of randomized clinical trials. eFigure 1 in the Supplement shows the results of all clinical trials in the analysis.

**Figure 3.**
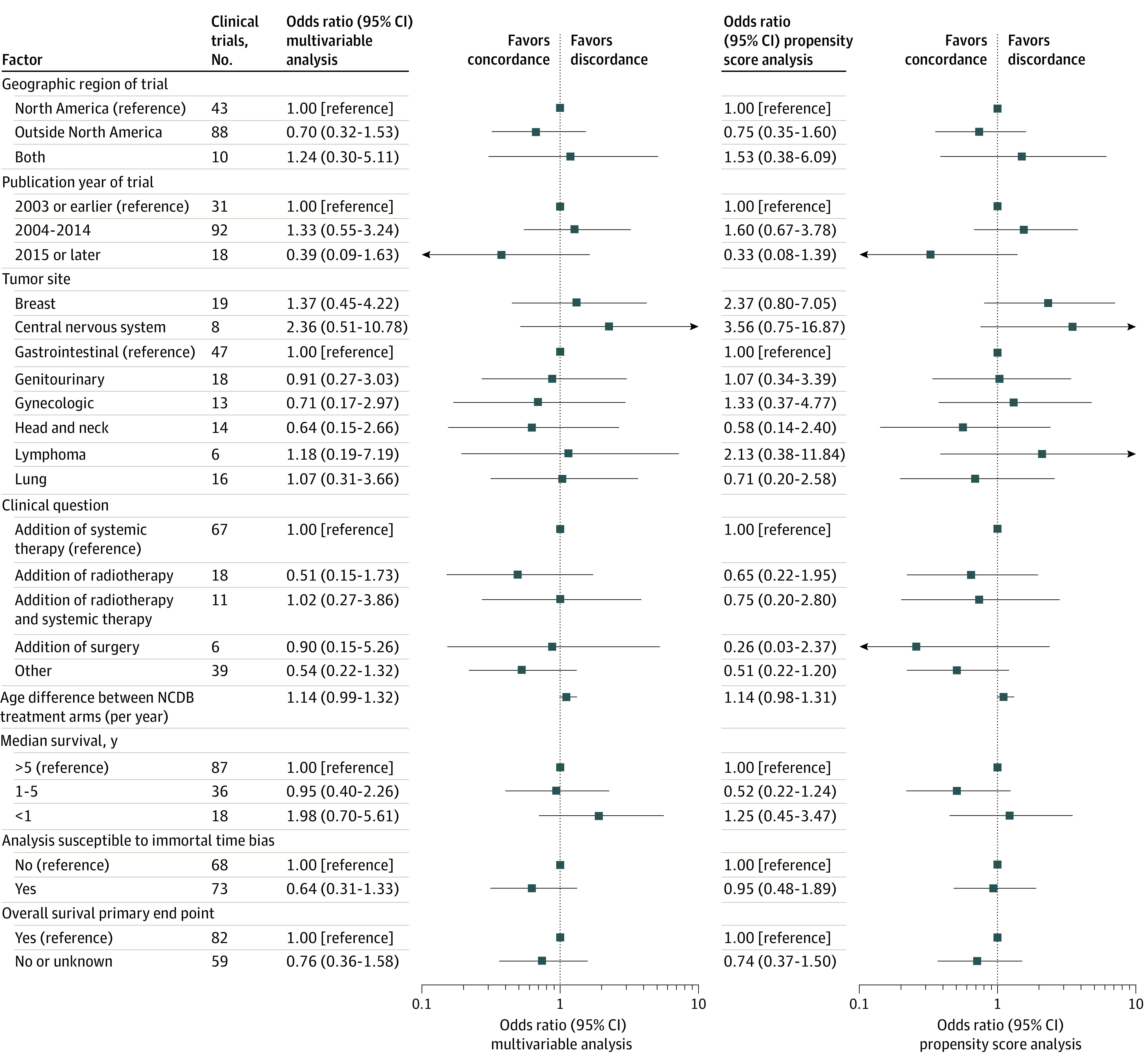
Estimators of Concordance in Hazard Ratios (HRs) With Randomized Clinical Trials and the National Cancer Database (NCDB) Univariable logistic regression analyses conducted to identify factors that estimate when an NCDB analysis will suggest results concordant with randomized clinical trials. Concordant NCDB analyses were defined as occasions when the NCDB HR fell within the 95% CI of the HR in the randomized clinical trial. Odds ratios less than 1 indicate that NCDB analyses were more likely to be concordant with clinical trials.

When evaluating concordance with statistical significance, we found that 58 trials (41%) that used univariable analysis, 65 trials (46%) that used multivariable analysis, and 63 trials (45%) that used propensity score analysis produced *P* values concordant with randomized clinical trials (Table 2). Forty-nine randomized clinical trials found one treatment arm to have significantly improved survival, yet NCDB analysis found nonsignificant *P* values or opposite findings in 21 trials (43%) analyzed with univariable analysis, 19 trials (39%) analyzed with multivariable analysis, and 19 trials (39%) analyzed with propensity score analysis. Ninety-two clinical trials found no significant difference between treatment arms, yet the NCDB found significant *P* values in 62 trials (67%) analyzed with univariable analysis, 57 trials (62%) analyzed with multivariable analysis, and 59 trials (64%) analyzed with propensity score analysis. eTable 2 in the Supplement reports whether clinical trial results agreed with NCDB analysis depending on whether the NCDB analysis significantly favored a more-aggressive or less-aggressive treatment arm. eFigure 2 in the Supplement shows the association between randomized clinical trial HRs and NCDB HRs when considering concordance with statistical significance. Analysis to identify individual estimators of concordance did not find any factors associated with concordance in HRs for overall survival between the NCDB and clinical trials (Figure 3).

**Table 2.** Concordance in Statistical Significance Between Clinical Trials and NCDB Analysis

Variable	Type of analysis, No. (%)		
	Univariable	Multivariable	Propensity score
**Clinical trial *P* value < .05 (n = 49)**			
NCDB analysis *P* < .05 with analysis			
Correctly favoring superior clinical trial arm	28 (57)	30 (61)	30 (61)
Incorrectly favoring inferior clinical trial arm	10 (20)	1 (2)	4 (8)
NCDB analysis *P* ≥ .05	11 (22)	18 (37)	15 (31)
**Clinical trial *P* value ≥ .05 (n = 92)**			
NCDB analysis			
*P* < .05	62 (67)	57 (62)	59 (64)
*P* ≥ .05	30 (33)	35 (38)	33 (36)

Abbreviation: NCDB, National Cancer Database.

## Discussion

This study noted a substantial lack of concordance and little correlation between survival outcomes of randomized clinical trials and comparative effectiveness research using cancer registry data. Multivariable models and propensity score analyses improved concordance modestly, yet approximately one-third of clinical trials replicated with cancer registry data produced overall survival outcomes discordant with those of clinical trials. Furthermore, this study did not identify factors associated with the clinical research question that might help to identify cancer registry research studies more or less in line with the results of randomized clinical trials.

These findings appear to complement those of other research in oncology^32^ and nononcology fields,^36,37,38^ which evaluated differences in outcomes between randomized clinical trials and observational research. Within the oncology setting, Soni et al^32^ reported a study using different methods comparing the results of published comparative effectiveness research with published randomized clinical trials. The study found that 62% of analyses with cancer registry data produced HRs for overall survival concordant with clinical trial data; this finding was similar to our study, which found concordance rates between 56% and 70% depending on the analytic method. Soni et al addressed the real-world application of cancer registry data by focusing on published research, whereas our study used a prespecified analytic approach. Overall, our study complements the investigation by Soni et al; together, the findings suggest that a large portion of comparative effectiveness research using cancer registry data may produce results that differ substantially from those of randomized clinical trials.

Our analysis of statistical significance found differences between observational cancer registry research and clinical trial outcomes, although one must consider that calculations of statistical significance depend on sample size. Therefore, the larger number of patients within cancer registries lead to a higher likelihood of a lower *P* value. In addition, publication bias with clinical research may lead to a decreased likelihood of studies reporting nonsignificant *P* values appearing in the literature. Therefore, the clinical trials assessed within our study may be skewed toward statistical significance. Research suggests that physicians frequently misunderstand the basic concepts of *P* values.^39,40^ Furthermore, the overall utility of the *P* value in research represents an active subject of debate.^41^ Despite these limitations, one must also accept the ubiquitous role of *P* values in clinical medicine. Clinical treatment decisions, drug approval, and policy recommendations often hinge on the question of statistical significance.^42,43,44^

Multiple factors could potentially explain the observed differences between comparative effectiveness research with registry data and randomized clinical trials, although selection bias represents a key factor. The lack of randomization in observational research suggests the possibility of selection bias, which represents an imbalance in known or unknown confounding factors between treatment groups. Variables such as body mass index, tobacco use, or performance status, which are not recorded in cancer registry data, could be factors in treatment decisions and independently affect survival regardless of the treatment chosen. One could hypothesize that more granular databases, such as cancer data linked to electronic health record data, could help better control for these confounding factors, although the role of these factors in study results deserves further examination.

Beyond selection bias, one must consider the potential influence of misclassified cancer registry data. The Charlson comorbidity index estimates a patient’s comorbidity, although research suggests that this index may provide only a rough measure of underlying comorbidity^45^ and additional research suggests that the Charlson comorbidity index score may be underreported within cancer registries.^46^ Similarly, research demonstrates the potential for underreporting of systemic therapy, hormonal therapy, and radiotherapy in cancer registry data.^8^ Overall, we lack a clear understanding of whether these misclassified variables occur at random, which would tend to bias results toward the null (ie, no survival difference) or occur not at random, which could be an unpredictable factor in survival analyses.

Beyond the aforementioned issues, one must consider that even with perfect cancer registry data, the outcomes between observational cancer registry research and randomized clinical trials might fundamentally differ. Patients in randomized clinical trials represent a younger group of individuals with better performance status and fewer comorbidities compared with the general oncology population.^47^ Differences in outcomes between clinical trials and comparative effectiveness research with registry data could potentially reflect a differential effect of treatment in healthier participants in randomized clinical trials.^48^ Similarly, one must consider the possibility that the technical delivery of cancer therapy in the real-world setting may differ from delivering cancer therapy under the constraints of a randomized clinical trial. In this case, one could potentially consider differences in observational data as a potential implementation failure as opposed to a true treatment failure. Overall, it is difficult to identify a single reason accounting for the differences in outcomes between observational research and randomized clinical trials. Ultimately, these differences in outcomes may stem from multiple contributing factors.

### Limitations

This study has limitations. We focused on analyzing concordance in overall survival and could not evaluate cancer-specific survival owing to the lack of cause of death information within the NCDB. While overall survival represents a common primary end point in randomized clinical trials, one could hypothesize that cancer-specific survival or cancer progression might represent end points more likely to produce concordant results between observational research studies and randomized clinical trials. This study focused on data within the NCDB because of the detailed treatment information provided with this data set. We cannot estimate whether the observed findings in this study hold with other observational data sets, including the Surveillance, Epidemiology, and End Results Program,^7^ administrative data sets, or more granular institution-specific data sets. This study attempted to recreate clinical trials with cancer registry data, although we could not account for all inclusion/exclusion criteria associated with clinical trials, including performance status, chemotherapy details (agents, dose, and schedule), hormone therapy agents, radiotherapy techniques, and specific details of surgery. This limitation holds true with our present analysis as well as any research involving cancer registry data. Furthermore, we could not account for differences in follow-up time between the NCDB and randomized clinical trials, which could potentially affect concordance. In addition, this study used a structured approach to analyzing cancer registry data, although one must acknowledge that incorporating different variables into our multivariable models or using different, more robust, propensity score analytic techniques could potentially affect these outcomes.

## Conclusions

The findings of this study suggest a substantial lack of concordance in survival outcomes between comparative effectiveness research with cancer registry data and randomized clinical trial outcomes. These discrepant findings could represent fundamental differences in patient selection or treatments rendered in randomized clinical trials compared with the real world or alternatively could stem from issues surrounding selection bias or misclassification of data within cancer registries. Regardless of the underlying cause, understanding these differences may help provide context to clinicians and researchers as they interpret comparative effectiveness research in oncology.

## Notes

Supplement. **eReferences.** All Randomized Clinical Trial References **eFigure 1.** Analysis of All Trials **eTable 1.** Examples of Clinical Trials Included and Excluded From Analysis **eTable 2.** Concordance in Statistical Significance Between Clinical Trials and NCDB Analysis Stratified by Whether the NCDB Analysis Favors More Aggressive Treatment Arm **eFigure 2.** Comparison of Hazard Ratios From Randomized Clinical Trials and Analyses With Data From NCDB
