# Supplementary material for: Evaluation of the Use of Cancer Registry Data for Comparative Effectiveness Research
Source: JAMA Netw Open. 2020 Jul 30;3(7):e2011985. doi: 10.1001/jamanetworkopen.2020.11985 (PMC9009816; doi:10.1001/jamanetworkopen.2020.11985)
Supplement: Supplement. — eReferences. All Randomized Clinical Trial References eFigure 1. Analysis of All Trials eTable 1. Examples of Clinical Trials Included and Excluded From Analysis eTable 2. Concordance in Statistical Significance Between Clinical Trials and NCDB Analysis Stratified by Whether the NCDB Analysis Favors More Aggressive Treatment Arm eFigure 2. Comparison of Hazard Ratios From Randomized Clinical Trials and Analyses With Data From NCDB [file jamanetwopen-e2011985-s001.pdf]

Supplementary Online Content

Kumar A, Guss ZD, Courtney PT, et al. Evaluation of the use of cancer registry data for comparative effectiveness research. *JAMA Netw Open*. 2020;3(7):e2011985.
doi:10.1001/jamanetworkopen.2020.11985

eReferences. All Randomized Clinical Trial References

eFigure 1. Analysis of All Trials

eTable 1. Examples of Clinical Trials Included and Excluded From Analysis

eTable 2. Concordance in Statistical Significance Between Clinical Trials and NCDB Analysis Stratified by Whether the NCDB Analysis Favors More Aggressive Treatment Arm

eFigure 2. Comparison of Hazard Ratios From Randomized Clinical Trials and Analyses With Data From NCDB

This supplementary material has been provided by the authors to give readers additional information about their work.

© 2020 Kumar A et al. *JAMA Network Open.*

eReferences. All randomized clinical trial references.

1. Ajani JA, Xiao L, Roth JA, et al. A phase II randomized trial of induction chemotherapy versus no induction chemotherapy followed by preoperative chemoradiation in patients with esophageal cancer. *Ann Oncol.* 2013;24(11):2844-2849.
2. Allum WH, Stenning SP, Bancewicz J, Clark PI, Langley RE. Long-term results of a randomized trial of surgery with or without preoperative chemotherapy in esophageal cancer. *J Clin Oncol.* 2009;27(30):5062-5067.
3. Arriagada R, Bergman B, Dunant A, et al. Cisplatin-based adjuvant chemotherapy in patients with completely resected non-small-cell lung cancer. *N Engl J Med.* 2004;350(4):351-360.
4. Bachaud JM, Cohen-Jonathan E, Alzieu C, David JM, Serrano E, Daly-Schveitzer N. Combined postoperative radiotherapy and weekly cisplatin infusion for locally advanced head and neck carcinoma: final report of a randomized trial. *Int J Radiat Oncol Biol Phys.* 1996;36(5):999-1004.
5. Bartelink H, Horiot JC, Poortmans PM, et al. Impact of a higher radiation dose on local control and survival in breast-conserving therapy of early breast cancer: 10-year results of the randomized boost versus no boost EORTC 22881-10882 trial. *J Clin Oncol.* 2007;25(22):3259-3265.
6. Bartelink H, Roelofsen F, Eschwege F, et al. Concomitant radiotherapy and chemotherapy is superior to radiotherapy alone in the treatment of locally advanced anal cancer: results of a phase III randomized trial of the European Organization for Research and Treatment of Cancer Radiotherapy and Gastrointestinal Cooperative Groups. *J Clin Oncol.* 1997;15(5):2040-2049.
7. Bellon JR, Come SE, Gelman RS, et al. Sequencing of chemotherapy and radiation therapy in early-stage breast cancer: updated results of a prospective randomized trial. *J Clin Oncol.* 2005;23(9):1934-1940.
8. Benedetti Panici P, Basile S, Maneschi F, et al. Systematic pelvic lymphadenectomy vs. no lymphadenectomy in early-stage endometrial carcinoma: randomized clinical trial. *J Natl Cancer Inst.* 2008;100(23):1707-1716.
9. Berlin JD, Catalano P, Thomas JP, Kugler JW, Haller DG, Benson AB, 3rd. Phase III study of gemcitabine in combination with fluorouracil versus gemcitabine alone in patients with advanced pancreatic carcinoma: Eastern Cooperative Oncology Group Trial E2297. *J Clin Oncol.* 2002;20(15):3270-3275.
10. Bernier J, Domenge C, Ozsahin M, et al. Postoperative irradiation with or without concomitant chemotherapy for locally advanced head and neck cancer. *N Engl J Med.* 2004;350(19):1945-1952.
11. Blichert-Toft M, Nielsen M, During M, et al. Long-term results of breast conserving surgery vs. mastectomy for early stage invasive breast cancer: 20-year follow-up of the Danish randomized DBCG-82TM protocol. *Acta Oncol.* 2008;47(4):672-681.
12. Bolla M, Van Tienhoven G, Warde P, et al. External irradiation with or without long-term androgen suppression for prostate cancer with high metastatic risk: 10-year results of an EORTC randomised study. *Lancet Oncol.* 2010;11(11):1066-1073.
13. Bonjer HJ, Deijen CL, Abis GA, et al. A randomized trial of laparoscopic versus open surgery for rectal cancer. *N Engl J Med.* 2015;372(14):1324-1332.
14. Bonner JA, Harari PM, Giralt J, et al. Radiotherapy plus cetuximab for locoregionally advanced head and neck cancer: 5-year survival data from a phase 3 randomised trial, and relation between cetuximab-induced rash and survival. *Lancet Oncol.* 2010;11(1):21-28.
15. Bonnet C, Fillet G, Mounier N, et al. CHOP alone compared with CHOP plus radiotherapy for localized aggressive lymphoma in elderly patients: a study by the Groupe d'Etude des Lymphomes de l'Adulte. *J Clin Oncol.* 2007;25(7):787-792.

© 2020 Kumar A et al. *JAMA Network Open.*

16. Bouche O, Ychou M, Burtin P, et al. Adjuvant chemotherapy with 5-fluorouracil and cisplatin compared with surgery alone for gastric cancer: 7-year results of the FFCD randomized phase III trial (8801). *Ann Oncol.* 2005;16(9):1488-1497.
17. Bozzetti F, Marubini E, Bonfanti G, Miceli R, Piano C, Gennari L. Subtotal versus total gastrectomy for gastric cancer: five-year survival rates in a multicenter randomized Italian trial. Italian Gastrointestinal Tumor Study Group. *Ann Surg.* 1999;230(2):170-178.
18. Browman GP, Cripps C, Hodson DI, Eapen L, Sathya J, Levine MN. Placebo-controlled randomized trial of infusional fluorouracil during standard radiotherapy in locally advanced head and neck cancer. *J Clin Oncol.* 1994;12(12):2648-2653.
19. Burmeister BH, Smithers BM, Gebski V, et al. Surgery alone versus chemoradiotherapy followed by surgery for resectable cancer of the oesophagus: a randomised controlled phase III trial. *Lancet Oncol.* 2005;6(9):659-668.
20. Burmeister BH, Thomas JM, Burmeister EA, et al. Is concurrent radiation therapy required in patients receiving preoperative chemotherapy for adenocarcinoma of the oesophagus? A randomised phase II trial. *Eur J Cancer.* 2011;47(3):354-360.
21. Butts CA, Ding K, Seymour L, et al. Randomized phase III trial of vinorelbine plus cisplatin compared with observation in completely resected stage IB and II non-small-cell lung cancer: updated survival analysis of JBR-10. *J Clin Oncol.* 2010;28(1):29-34.
22. Chauffert B, Mornex F, Bonnetain F, et al. Phase III trial comparing intensive induction chemoradiotherapy (60 Gy, infusional 5-FU and intermittent cisplatin) followed by maintenance gemcitabine with gemcitabine alone for locally advanced unresectable pancreatic cancer. Definitive results of the 2000-01 FFCD/SFRO study. *Ann Oncol.* 2008;19(9):1592-1599.
23. Cohen EE, Karrison TG, Kocherginsky M, et al. Phase III randomized trial of induction chemotherapy in patients with N2 or N3 locally advanced head and neck cancer. *J Clin Oncol.* 2014;32(25):2735-2743.
24. Coiffier B, Thieblemont C, Van Den Neste E, et al. Long-term outcome of patients in the LNH-98.5 trial, the first randomized study comparing rituximab-CHOP to standard CHOP chemotherapy in DLBCL patients: a study by the Groupe d'Etudes des Lymphomes de l'Adulte. *Blood.* 2010;116(12):2040-2045.
25. Colucci G, Labianca R, Di Costanzo F, et al. Randomized phase III trial of gemcitabine plus cisplatin compared with single-agent gemcitabine as first-line treatment of patients with advanced pancreatic cancer: the GIP-1 study. *J Clin Oncol.* 2010;28(10):1645-1651.
26. Conroy T, Desseigne F, Ychou M, et al. FOLFIRINOX versus gemcitabine for metastatic pancreatic cancer. *N Engl J Med.* 2011;364(19):1817-1825.
27. Cooper JS, Zhang Q, Pajak TF, et al. Long-term follow-up of the RTOG 9501/intergroup phase III trial: postoperative concurrent radiation therapy and chemotherapy in high-risk squamous cell carcinoma of the head and neck. *Int J Radiat Oncol Biol Phys.* 2012;84(5):1198-1205.
28. Creutzberg CL, Nout RA, Lybeert ML, et al. Fifteen-year radiotherapy outcomes of the randomized PORTEC-1 trial for endometrial carcinoma. *Int J Radiat Oncol Biol Phys.* 2011;81(4):e631-638.
29. Cunningham D, Allum WH, Stenning SP, et al. Perioperative chemotherapy versus surgery alone for resectable gastroesophageal cancer. *N Engl J Med.* 2006;355(1):11-20.
30. Cunningham D, Chau I, Stocken DD, et al. Phase III randomized comparison of gemcitabine versus gemcitabine plus capecitabine in patients with advanced pancreatic cancer. *J Clin Oncol.* 2009;27(33):5513-5518.
31. D'Amico AV, Chen MH, Renshaw AA, Loffredo M, Kantoff PW. Androgen suppression and radiation vs radiation alone for prostate cancer: a randomized trial. *JAMA.* 2008;299(3):289-295.
32. De Vita F, Giuliani F, Orditura M, et al. Adjuvant chemotherapy with epirubicin, leucovorin, 5-fluorouracil and etoposide regimen in resected gastric cancer patients: a randomized phase III trial by the Gruppo Oncologico Italia Meridionale (GOIM 9602 Study). *Ann Oncol.* 2007;18(8):1354-1358.

© 2020 Kumar A et al. *JAMA Network Open.*

33. Dearnaley DP, Jovic G, Syndikus I, et al. Escalated-dose versus control-dose conformal radiotherapy for prostate cancer: long-term results from the MRC RT01 randomised controlled trial. *Lancet Oncol.* 2014;15(4):464-473.
34. Denis F, Garaud P, Bardet E, et al. Final results of the 94-01 French Head and Neck Oncology and Radiotherapy Group randomized trial comparing radiotherapy alone with concomitant radiochemotherapy in advanced-stage oropharynx carcinoma. *J Clin Oncol.* 2004;22(1):69-76.
35. Depierre A, Milleron B, Moro-Sibilot D, et al. Preoperative chemotherapy followed by surgery compared with primary surgery in resectable stage I (except T1N0), II, and IIIa non-small-cell lung cancer. *J Clin Oncol.* 2002;20(1):247-253.
36. Di Costanzo F, Gasperoni S, Manzione L, et al. Adjuvant chemotherapy in completely resected gastric cancer: a randomized phase III trial conducted by GOIRC. *J Natl Cancer Inst.* 2008;100(6):388-398.
37. Dillman RO, Herndon J, Seagren SL, Eaton WL, Jr., Green MR. Improved survival in stage III non-small-cell lung cancer: seven-year follow-up of cancer and leukemia group B (CALGB) 8433 trial. *J Natl Cancer Inst.* 1996;88(17):1210-1215.
38. Douillard JY, Rosell R, De Lena M, et al. Adjuvant vinorelbine plus cisplatin versus observation in patients with completely resected stage IB-IIIA non-small-cell lung cancer (Adjuvant Navelbine International Trialist Association [ANITA]): a randomised controlled trial. *Lancet Oncol.* 2006;7(9):719-727.
39. Duenas-Gonzalez A, Zarba JJ, Patel F, et al. Phase III, open-label, randomized study comparing concurrent gemcitabine plus cisplatin and radiation followed by adjuvant gemcitabine and cisplatin versus concurrent cisplatin and radiation in patients with stage IIB to IVA carcinoma of the cervix. *J Clin Oncol.* 2011;29(13):1678-1685.
40. Eifel PJ, Winter K, Morris M, et al. Pelvic irradiation with concurrent chemotherapy versus pelvic and para-aortic irradiation for high-risk cervical cancer: an update of radiation therapy oncology group trial (RTOG) 90-01. *J Clin Oncol.* 2004;22(5):872-880.
41. Engert A, Franklin J, Eich HT, et al. Two cycles of doxorubicin, bleomycin, vinblastine, and dacarbazine plus extended-field radiotherapy is superior to radiotherapy alone in early favorable Hodgkin's lymphoma: final results of the GHSG HD7 trial. *J Clin Oncol.* 2007;25(23):3495-3502.
42. Eschwege F, Sancho-Garnier H, Gerard JP, et al. Ten-year results of randomized trial comparing radiotherapy and concomitant bleomycin to radiotherapy alone in epidermoid carcinomas of the oropharynx: experience of the European Organization for Research and Treatment of Cancer. *NCI Monogr.* 1988(6):275-278.
43. Faivre-Finn C, Snee M, Ashcroft L, et al. Concurrent once-daily versus twice-daily chemoradiotherapy in patients with limited-stage small-cell lung cancer (CONVERT): an open-label, phase 3, randomised, superiority trial. *Lancet Oncol.* 2017;18(8):1116-1125.
44. Feng YR, Zhu Y, Liu LY, et al. Interim analysis of postoperative chemoradiotherapy with capecitabine and oxaliplatin versus capecitabine alone for pathological stage II and III rectal cancer: a randomized multicenter phase III trial. *Oncotarget.* 2016;7(18):25576-25584.
45. Fernandez-Martos C, Garcia-Albeniz X, Pericay C, et al. Chemoradiation, surgery and adjuvant chemotherapy versus induction chemotherapy followed by chemoradiation and surgery: long-term results of the Spanish GCR-3 phase II randomized trialdagger. *Ann Oncol.* 2015;26(8):1722-1728.
46. Fisher B, Dignam J, Wolmark N, et al. Lumpectomy and radiation therapy for the treatment of intraductal breast cancer: findings from National Surgical Adjuvant Breast and Bowel Project B-17. *J Clin Oncol.* 1998;16(2):441-452.
47. Gerard JP, Conroy T, Bonnetain F, et al. Preoperative radiotherapy with or without concurrent fluorouracil and leucovorin in T3-4 rectal cancers: results of FFCD 9203. *J Clin Oncol.* 2006;24(28):4620-4625.

© 2020 Kumar A et al. *JAMA Network Open.*

48. Giuliano AE, Hunt KK, Ballman KV, et al. Axillary dissection vs no axillary dissection in women with invasive breast cancer and sentinel node metastasis: a randomized clinical trial. *JAMA.* 2011;305(6):569-575.
49. Goldstein D, El-Maraghi RH, Hammel P, et al. nab-Paclitaxel plus gemcitabine for metastatic pancreatic cancer: long-term survival from a phase III trial. *J Natl Cancer Inst.* 2015;107(2).
50. Gravis G, Boher JM, Joly F, et al. Androgen Deprivation Therapy (ADT) Plus Docetaxel Versus ADT Alone in Metastatic Non castrate Prostate Cancer: Impact of Metastatic Burden and Long-term Survival Analysis of the Randomized Phase 3 GETUG-AFU15 Trial. *Eur Urol.* 2016;70(2):256-262.
51. Group ST, Bentzen SM, Agrawal RK, et al. The UK Standardisation of Breast Radiotherapy (START) Trial B of radiotherapy hypofractionation for treatment of early breast cancer: a randomised trial. *Lancet.* 2008;371(9618):1098-1107.
52. Haddad R, O'Neill A, Rabinowits G, et al. Induction chemotherapy followed by concurrent chemoradiotherapy (sequential chemoradiotherapy) versus concurrent chemoradiotherapy alone in locally advanced head and neck cancer (PARADIGM): a randomised phase 3 trial. *Lancet Oncol.* 2013;14(3):257-264.
53. Heinemann V, Quietzsch D, Gieseler F, et al. Randomized phase III trial of gemcitabine plus cisplatin compared with gemcitabine alone in advanced pancreatic cancer. *J Clin Oncol.* 2006;24(24):3946-3952.
54. Herrmann R, Bodoky G, Ruhstaller T, et al. Gemcitabine plus capecitabine compared with gemcitabine alone in advanced pancreatic cancer: a randomized, multicenter, phase III trial of the Swiss Group for Clinical Cancer Research and the Central European Cooperative Oncology Group. *J Clin Oncol.* 2007;25(16):2212-2217.
55. Hoskin PJ, Rojas AM, Bownes PJ, Lowe GJ, Ostler PJ, Bryant L. Randomised trial of external beam radiotherapy alone or combined with high-dose-rate brachytherapy boost for localised prostate cancer. *Radiother Oncol.* 2012;103(2):217-222.
56. Hui EP, Ma BB, Leung SF, et al. Randomized phase II trial of concurrent cisplatin-radiotherapy with or without neoadjuvant docetaxel and cisplatin in advanced nasopharyngeal carcinoma. *J Clin Oncol.* 2009;27(2):242-249.
57. Huscher CG, Mingoli A, Sgarzini G, et al. Laparoscopic versus open subtotal gastrectomy for distal gastric cancer: five-year results of a randomized prospective trial. *Ann Surg.* 2005;241(2):232-237.
58. International Collaboration of T, Medical Research Council Advanced Bladder Cancer Working P, European Organisation for R, et al. International phase III trial assessing neoadjuvant cisplatin, methotrexate, and vinblastine chemotherapy for muscle-invasive bladder cancer: long-term results of the BA06 30894 trial. *J Clin Oncol.* 2011;29(16):2171-2177.
59. Janda M, Gebski V, Davies LC, et al. Effect of Total Laparoscopic Hysterectomy vs Total Abdominal Hysterectomy on Disease-Free Survival Among Women With Stage I Endometrial Cancer: A Randomized Clinical Trial. *JAMA.* 2017;317(12):1224-1233.
60. Jeong SY, Park JW, Nam BH, et al. Open versus laparoscopic surgery for mid-rectal or low-rectal cancer after neoadjuvant chemoradiotherapy (COREAN trial): survival outcomes of an open-label, non-inferiority, randomised controlled trial. *Lancet Oncol.* 2014;15(7):767-774.
61. Jones CU, Hunt D, McGowan DG, et al. Radiotherapy and short-term androgen deprivation for localized prostate cancer. *N Engl J Med.* 2011;365(2):107-118.
62. Karim AB, Maat B, Hatlevoll R, et al. A randomized trial on dose-response in radiation therapy of low-grade cerebral glioma: European Organization for Research and Treatment of Cancer (EORTC) Study 22844. *Int J Radiat Oncol Biol Phys.* 1996;36(3):549-556.
63. Kaufmann M, Jonat W, Blamey R, et al. Survival analyses from the ZEBRA study. goserelin (Zoladex) versus CMF in premenopausal women with node-positive breast cancer. *Eur J Cancer.* 2003;39(12):1711-1717.

© 2020 Kumar A et al. *JAMA Network Open.*

64. Keime-Guibert F, Chinot O, Taillandier L, et al. Radiotherapy for glioblastoma in the elderly. *N Engl J Med.* 2007;356(15):1527-1535.
65. Keller SM, Adak S, Wagner H, et al. A randomized trial of postoperative adjuvant therapy in patients with completely resected stage II or IIIA non-small-cell lung cancer. Eastern Cooperative Oncology Group. *N Engl J Med.* 2000;343(17):1217-1222.
66. Kelsen DP, Winter KA, Gunderson LL, et al. Long-term results of RTOG trial 8911 (USA Intergroup 113): a random assignment trial comparison of chemotherapy followed by surgery compared with surgery alone for esophageal cancer. *J Clin Oncol.* 2007;25(24):3719-3725.
67. Keys HM, Roberts JA, Brunetto VL, et al. A phase III trial of surgery with or without adjunctive external pelvic radiation therapy in intermediate risk endometrial adenocarcinoma: a Gynecologic Oncology Group study. *Gynecol Oncol.* 2004;92(3):744-751.
68. Krag DN, Anderson SJ, Julian TB, et al. Sentinel-lymph-node resection compared with conventional axillary-lymph-node dissection in clinically node-negative patients with breast cancer: overall survival findings from the NSABP B-32 randomised phase 3 trial. *Lancet Oncol.* 2010;11(10):927-933.
69. Kuban DA, Tucker SL, Dong L, et al. Long-term results of the M. D. Anderson randomized dose-escalation trial for prostate cancer. *Int J Radiat Oncol Biol Phys.* 2008;70(1):67-74.
70. Kulig J, Kolodziejczyk P, Sierzega M, et al. Adjuvant chemotherapy with etoposide, adriamycin and cisplatin compared with surgery alone in the treatment of gastric cancer: a phase III randomized, multicenter, clinical trial. *Oncology.* 2010;78(1):54-61.
71. Kunkler IH, Williams LJ, Jack WJ, Cameron DA, Dixon JM, investigators PI. Breast-conserving surgery with or without irradiation in women aged 65 years or older with early breast cancer (PRIME II): a randomised controlled trial. *Lancet Oncol.* 2015;16(3):266-273.
72. Landoni F, Maneo A, Colombo A, et al. Randomised study of radical surgery versus radiotherapy for stage Ib-IIa cervical cancer. *Lancet.* 1997;350(9077):535-540.
73. Le Chevalier T, Arriagada R, Quoix E, et al. Radiotherapy alone versus combined chemotherapy and radiotherapy in nonresectable non-small-cell lung cancer: first analysis of a randomized trial in 353 patients. *J Natl Cancer Inst.* 1991;83(6):417-423.
74. Lee WR, Dignam JJ, Amin MB, et al. Randomized Phase III Noninferiority Study Comparing Two Radiotherapy Fractionation Schedules in Patients With Low-Risk Prostate Cancer. *J Clin Oncol.* 2016;34(20):2325-2332.
75. Lehmann J, Franzaring L, Thuroff J, Wellek S, Stockle M. Complete long-term survival data from a trial of adjuvant chemotherapy vs control after radical cystectomy for locally advanced bladder cancer. *BJU Int.* 2006;97(1):42-47.
76. Litiere S, Werutsky G, Fentiman IS, et al. Breast conserving therapy versus mastectomy for stage I-II breast cancer: 20 year follow-up of the EORTC 10801 phase 3 randomised trial. *Lancet Oncol.* 2012;13(4):412-419.
77. Loehrer PJ, Sr., Feng Y, Cardenes H, et al. Gemcitabine alone versus gemcitabine plus radiotherapy in patients with locally advanced pancreatic cancer: an Eastern Cooperative Oncology Group trial. *J Clin Oncol.* 2011;29(31):4105-4112.
78. Louvet C, Labianca R, Hammel P, et al. Gemcitabine in combination with oxaliplatin compared with gemcitabine alone in locally advanced or metastatic pancreatic cancer: results of a GERCOR and GISCAD phase III trial. *J Clin Oncol.* 2005;23(15):3509-3516.
79. Magrini SM, Buglione M, Corvo R, et al. Cetuximab and Radiotherapy Versus Cisplatin and Radiotherapy for Locally Advanced Head and Neck Cancer: A Randomized Phase II Trial. *J Clin Oncol.* 2016;34(5):427-435.
80. Marcus R, Imrie K, Solal-Celigny P, et al. Phase III study of R-CVP compared with cyclophosphamide, vincristine, and prednisone alone in patients with previously untreated advanced follicular lymphoma. *J Clin Oncol.* 2008;26(28):4579-4586.

© 2020 Kumar A et al. *JAMA Network Open.*

81. Mariette C, Dahan L, Mornex F, et al. Surgery alone versus chemoradiotherapy followed by surgery for stage I and II esophageal cancer: final analysis of randomized controlled phase III trial FFCD 9901. *J Clin Oncol.* 2014;32(23):2416-2422.
82. Mason MD, Parulekar WR, Sydes MR, et al. Final Report of the Intergroup Randomized Study of Combined Androgen-Deprivation Therapy Plus Radiotherapy Versus Androgen-Deprivation Therapy Alone in Locally Advanced Prostate Cancer. *J Clin Oncol.* 2015;33(19):2143-2150.
83. McCormick B, Winter K, Hudis C, et al. RTOG 9804: a prospective randomized trial for good-risk ductal carcinoma in situ comparing radiotherapy with observation. *J Clin Oncol.* 2015;33(7):709-715.
84. McLeod DG, See WA, Klimberg I, et al. The bicalutamide 150 mg early prostate cancer program: findings of the North American trial at 7.7-year median followup. *J Urol.* 2006;176(1):75-80.
85. Medical Research Council Brain Tumor Working P. Randomized trial of procarbazine, lomustine, and vincristine in the adjuvant treatment of high-grade astrocytoma: a Medical Research Council trial. *J Clin Oncol.* 2001;19(2):509-518.
86. Millikan R, Dinney C, Swanson D, et al. Integrated therapy for locally advanced bladder cancer: final report of a randomized trial of cystectomy plus adjuvant M-VAC versus cystectomy with both preoperative and postoperative M-VAC. *J Clin Oncol.* 2001;19(20):4005-4013.
87. Minsky BD, Pajak TF, Ginsberg RJ, et al. INT 0123 (Radiation Therapy Oncology Group 94-05) phase III trial of combined-modality therapy for esophageal cancer: high-dose versus standard-dose radiation therapy. *J Clin Oncol.* 2002;20(5):1167-1174.
88. Murray N, Coy P, Pater JL, et al. Importance of timing for thoracic irradiation in the combined modality treatment of limited-stage small-cell lung cancer. The National Cancer Institute of Canada Clinical Trials Group. *J Clin Oncol.* 1993;11(2):336-344.
89. Muss HB, Berry DA, Cirrincione CT, et al. Adjuvant chemotherapy in older women with early-stage breast cancer. *N Engl J Med.* 2009;360(20):2055-2065.
90. Nakajima T, Nashimoto A, Kitamura M, et al. Adjuvant mitomycin and fluorouracil followed by oral uracil plus tegafur in serosa-negative gastric cancer: a randomised trial. Gastric Cancer Surgical Study Group. *Lancet.* 1999;354(9175):273-277.
91. Neoptolemos JP, Palmer DH, Ghaneh P, et al. Comparison of adjuvant gemcitabine and capecitabine with gemcitabine monotherapy in patients with resected pancreatic cancer (ESPAC-4): a multicentre, open-label, randomised, phase 3 trial. *Lancet.* 2017;389(10073):1011-1024.
92. Nguyen-Tan PF, Zhang Q, Ang KK, et al. Randomized phase III trial to test accelerated versus standard fractionation in combination with concurrent cisplatin for head and neck carcinomas in the Radiation Therapy Oncology Group 0129 trial: long-term report of efficacy and toxicity. *J Clin Oncol.* 2014;32(34):3858-3866.
93. Northover J, Glynne-Jones R, Sebag-Montefiore D, et al. Chemoradiation for the treatment of epidermoid anal cancer: 13-year follow-up of the first randomised UKCCCR Anal Cancer Trial (ACT I). *Br J Cancer.* 2010;102(7):1123-1128.
94. Nout RA, Smit VT, Putter H, et al. Vaginal brachytherapy versus pelvic external beam radiotherapy for patients with endometrial cancer of high-intermediate risk (PORTEC-2): an open-label, non-inferiority, randomised trial. *Lancet.* 2010;375(9717):816-823.
95. Oettle H, Neuhaus P, Hochhaus A, et al. Adjuvant chemotherapy with gemcitabine and long-term outcomes among patients with resected pancreatic cancer: the CONKO-001 randomized trial. *JAMA.* 2013;310(14):1473-1481.
96. Overgaard M, Hansen PS, Overgaard J, et al. Postoperative radiotherapy in high-risk premenopausal women with breast cancer who receive adjuvant chemotherapy. Danish Breast Cancer Cooperative Group 82b Trial. *N Engl J Med.* 1997;337(14):949-955.
97. Overgaard M, Jensen MB, Overgaard J, et al. Postoperative radiotherapy in high-risk postmenopausal breast-cancer patients given adjuvant tamoxifen: Danish Breast Cancer Cooperative Group DBCG 82c randomised trial. *Lancet.* 1999;353(9165):1641-1648.

© 2020 Kumar A et al. *JAMA Network Open.*

98. Pfreundschuh M, Kuhnt E, Trumper L, et al. CHOP-like chemotherapy with or without rituximab in young patients with good-prognosis diffuse large-B-cell lymphoma: 6-year results of an open-label randomised study of the MabThera International Trial (MInT) Group. *Lancet Oncol.* 2011;12(11):1013-1022.
99. Pisters KM, Vallieres E, Crowley JJ, et al. Surgery with or without preoperative paclitaxel and carboplatin in early-stage non-small-cell lung cancer: Southwest Oncology Group Trial S9900, an intergroup, randomized, phase III trial. *J Clin Oncol.* 2010;28(11):1843-1849.
100. Quoix E, Zalcman G, Oster JP, et al. Carboplatin and weekly paclitaxel doublet chemotherapy compared with monotherapy in elderly patients with advanced non-small-cell lung cancer: IFCT-0501 randomised, phase 3 trial. *Lancet.* 2011;378(9796):1079-1088.
101. Randall ME, Filiaci VL, Muss H, et al. Randomized phase III trial of whole-abdominal irradiation versus doxorubicin and cisplatin chemotherapy in advanced endometrial carcinoma: a Gynecologic Oncology Group Study. *J Clin Oncol.* 2006;24(1):36-44.
102. Reed NS, Mangioni C, Malmstrom H, et al. Phase III randomised study to evaluate the role of adjuvant pelvic radiotherapy in the treatment of uterine sarcomas stages I and II: an European Organisation for Research and Treatment of Cancer Gynaecological Cancer Group Study (protocol 55874). *Eur J Cancer.* 2008;44(6):808-818.
103. Reni M, Cordio S, Milandri C, et al. Gemcitabine versus cisplatin, epirubicin, fluorouracil, and gemcitabine in advanced pancreatic cancer: a randomised controlled multicentre phase III trial. *Lancet Oncol.* 2005;6(6):369-376.
104. Roa W, Brasher PM, Bauman G, et al. Abbreviated course of radiation therapy in older patients with glioblastoma multiforme: a prospective randomized clinical trial. *J Clin Oncol.* 2004;22(9):1583-1588.
105. Roach M, 3rd, Bae K, Speight J, et al. Short-term neoadjuvant androgen deprivation therapy and external-beam radiotherapy for locally advanced prostate cancer: long-term results of RTOG 8610. *J Clin Oncol.* 2008;26(4):585-591.
106. Robert N, Leyland-Jones B, Asmar L, et al. Randomized phase III study of trastuzumab, paclitaxel, and carboplatin compared with trastuzumab and paclitaxel in women with HER-2-overexpressing metastatic breast cancer. *J Clin Oncol.* 2006;24(18):2786-2792.
107. Rocha Lima CM, Green MR, Rotche R, et al. Irinotecan plus gemcitabine results in no survival advantage compared with gemcitabine monotherapy in patients with locally advanced or metastatic pancreatic cancer despite increased tumor response rate. *J Clin Oncol.* 2004;22(18):3776-3783.
108. Rosell R, Gomez-Codina J, Camps C, et al. Preresectional chemotherapy in stage IIIA non-small-cell lung cancer: a 7-year assessment of a randomized controlled trial. *Lung Cancer.* 1999;26(1):7-14.
109. Roth JA, Fossella F, Komaki R, et al. A randomized trial comparing perioperative chemotherapy and surgery with surgery alone in resectable stage IIIA non-small-cell lung cancer. *J Natl Cancer Inst.* 1994;86(9):673-680.
110. Sasako M, Sakuramoto S, Katai H, et al. Five-year outcomes of a randomized phase III trial comparing adjuvant chemotherapy with S-1 versus surgery alone in stage II or III gastric cancer. *J Clin Oncol.* 2011;29(33):4387-4393.
111. Sathya JR, Davis IR, Julian JA, et al. Randomized trial comparing iridium implant plus external-beam radiation therapy with external-beam radiation therapy alone in node-negative locally advanced cancer of the prostate. *J Clin Oncol.* 2005;23(6):1192-1199.
112. Sauer R, Liersch T, Merkel S, et al. Preoperative versus postoperative chemoradiotherapy for locally advanced rectal cancer: results of the German CAO/ARO/AIO-94 randomized phase III trial after a median follow-up of 11 years. *J Clin Oncol.* 2012;30(16):1926-1933.
113. Scagliotti GV, Pastorino U, Vansteenkiste JF, et al. Randomized phase III study of surgery alone or surgery plus preoperative cisplatin and gemcitabine in stages IB to IIIA non-small-cell lung cancer. *J Clin Oncol.* 2012;30(2):172-178.

114. Shaw EG, Wang M, Coons SW, et al. Randomized trial of radiation therapy plus procarbazine, lomustine, and vincristine chemotherapy for supratentorial adult low-grade glioma: initial results of RTOG 9802. *J Clin Oncol.* 2012;30(25):3065-3070.
115. Smalley SR, Benedetti JK, Haller DG, et al. Updated analysis of SWOG-directed intergroup study 0116: a phase III trial of adjuvant radiochemotherapy versus observation after curative gastric cancer resection. *J Clin Oncol.* 2012;30(19):2327-2333.
116. Smeenk HG, van Eijck CH, Hop WC, et al. Long-term survival and metastatic pattern of pancreatic and periampullary cancer after adjuvant chemoradiation or observation: long-term results of EORTC trial 40891. *Ann Surg.* 2007;246(5):734-740.
117. Souhami RL, Spiro SG, Rudd RM, et al. Five-day oral etoposide treatment for advanced small-cell lung cancer: randomized comparison with intravenous chemotherapy. *J Natl Cancer Inst.* 1997;89(8):577-580.
118. Stathopoulos GP, Syrigos K, Aravantinos G, et al. A multicenter phase III trial comparing irinotecan-gemcitabine (IG) with gemcitabine (G) monotherapy as first-line treatment in patients with locally advanced or metastatic pancreatic cancer. *Br J Cancer.* 2006;95(5):587-592.
119. Stupp R, Hegi ME, Mason WP, et al. Effects of radiotherapy with concomitant and adjuvant temozolomide versus radiotherapy alone on survival in glioblastoma in a randomised phase III study: 5-year analysis of the EORTC-NCIC trial. *Lancet Oncol.* 2009;10(5):459-466.
120. Sun Y, Li WF, Chen NY, et al. Induction chemotherapy plus concurrent chemoradiotherapy versus concurrent chemoradiotherapy alone in locoregionally advanced nasopharyngeal carcinoma: a phase 3, multicentre, randomised controlled trial. *Lancet Oncol.* 2016;17(11):1509-1520.
121. Sweeney CJ, Chen YH, Carducci M, et al. Chemohormonal Therapy in Metastatic Hormone-Sensitive Prostate Cancer. *N Engl J Med.* 2015;373(8):737-746.
122. Tepper J, Krasna MJ, Niedzwiecki D, et al. Phase III trial of trimodality therapy with cisplatin, fluorouracil, radiotherapy, and surgery compared with surgery alone for esophageal cancer: CALGB 9781. *J Clin Oncol.* 2008;26(7):1086-1092.
123. Thatcher N, Hirsch FR, Luft AV, et al. Necitumumab plus gemcitabine and cisplatin versus gemcitabine and cisplatin alone as first-line therapy in patients with stage IV squamous non-small-cell lung cancer (SQUIRE): an open-label, randomised, controlled phase 3 trial. *Lancet Oncol.* 2015;16(7):763-774.
124. Thiel E, Korfel A, Martus P, et al. High-dose methotrexate with or without whole brain radiotherapy for primary CNS lymphoma (G-PCNSL-SG-1): a phase 3, randomised, non-inferiority trial. *Lancet Oncol.* 2010;11(11):1036-1047.
125. Thompson IM, Tangen CM, Paradelo J, et al. Adjuvant radiotherapy for pathological T3N0M0 prostate cancer significantly reduces risk of metastases and improves survival: long-term followup of a randomized clinical trial. *J Urol.* 2009;181(3):956-962.
126. Urba SG, Orringer MB, Turrisi A, Iannettoni M, Forastiere A, Strawderman M. Randomized trial of preoperative chemoradiation versus surgery alone in patients with locoregional esophageal carcinoma. *J Clin Oncol.* 2001;19(2):305-313.
127. van den Bent MJ, Brandes AA, Taphoorn MJ, et al. Adjuvant procarbazine, lomustine, and vincristine chemotherapy in newly diagnosed anaplastic oligodendroglioma: long-term follow-up of EORTC brain tumor group study 26951. *J Clin Oncol.* 2013;31(3):344-350.
128. van Hagen P, Hulshof MC, van Lanschot JJ, et al. Preoperative chemoradiotherapy for esophageal or junctional cancer. *N Engl J Med.* 2012;366(22):2074-2084.
129. Van Laethem JL, Hammel P, Mornex F, et al. Adjuvant gemcitabine alone versus gemcitabine-based chemoradiotherapy after curative resection for pancreatic cancer: a randomized EORTC-40013-22012/FFCD-9203/GERCOR phase II study. *J Clin Oncol.* 2010;28(29):4450-4456.
130. Veronesi U, Cascinelli N, Mariani L, et al. Twenty-year follow-up of a randomized study comparing breast-conserving surgery with radical mastectomy for early breast cancer. *N Engl J Med.* 2002;347(16):1227-1232.

© 2020 Kumar A et al. *JAMA Network Open.*

131. Veronesi U, Paganelli G, Viale G, et al. A randomized comparison of sentinel-node biopsy with routine axillary dissection in breast cancer. *N Engl J Med.* 2003;349(6):546-553.
132. Walker JL, Piedmonte MR, Spirtos NM, et al. Recurrence and survival after random assignment to laparoscopy versus laparotomy for comprehensive surgical staging of uterine cancer: Gynecologic Oncology Group LAP2 Study. *J Clin Oncol.* 2012;30(7):695-700.
133. Walsh TN, Noonan N, Hollywood D, Kelly A, Keeling N, Hennessy TP. A comparison of multimodal therapy and surgery for esophageal adenocarcinoma. *N Engl J Med.* 1996;335(7):462-467.
134. Wendt TG, Grabenbauer GG, Rodel CM, et al. Simultaneous radiochemotherapy versus radiotherapy alone in advanced head and neck cancer: a randomized multicenter study. *J Clin Oncol.* 1998;16(4):1318-1324.
135. Whelan TJ, Olivotto IA, Parulekar WR, et al. Regional Nodal Irradiation in Early-Stage Breast Cancer. *N Engl J Med.* 2015;373(4):307-316.
136. Whelan TJ, Pignol JP, Levine MN, et al. Long-term results of hypofractionated radiation therapy for breast cancer. *N Engl J Med.* 2010;362(6):513-520.
137. Wick W, Platten M, Meisner C, et al. Temozolomide chemotherapy alone versus radiotherapy alone for malignant astrocytoma in the elderly: the NOA-08 randomised, phase 3 trial. *Lancet Oncol.* 2012;13(7):707-715.
138. Widmark A, Klepp O, Solberg A, et al. Endocrine treatment, with or without radiotherapy, in locally advanced prostate cancer (SPCG-7/SFUO-3): an open randomised phase III trial. *Lancet.* 2009;373(9660):301-308.
139. Wolfson AH, Brady MF, Rocereto T, et al. A gynecologic oncology group randomized phase III trial of whole abdominal irradiation (WAI) vs. cisplatin-ifosfamide and mesna (CIM) as post-surgical therapy in stage I-IV carcinosarcoma (CS) of the uterus. *Gynecol Oncol.* 2007;107(2):177-185.
140. Wong LC, Ngan HY, Cheung AN, Cheng DK, Ng TY, Choy DT. Chemoradiation and adjuvant chemotherapy in cervical cancer. *J Clin Oncol.* 1999;17(7):2055-2060.
141. Ychou M, Boige V, Pignon JP, et al. Perioperative chemotherapy compared with surgery alone for resectable gastroesophageal adenocarcinoma: an FNCLCC and FFCD multicenter phase III trial. *J Clin Oncol.* 2011;29(13):1715-1721.

© 2020 Kumar A et al. *JAMA Network Open.*

eFigure 1. Analysis of all trials.

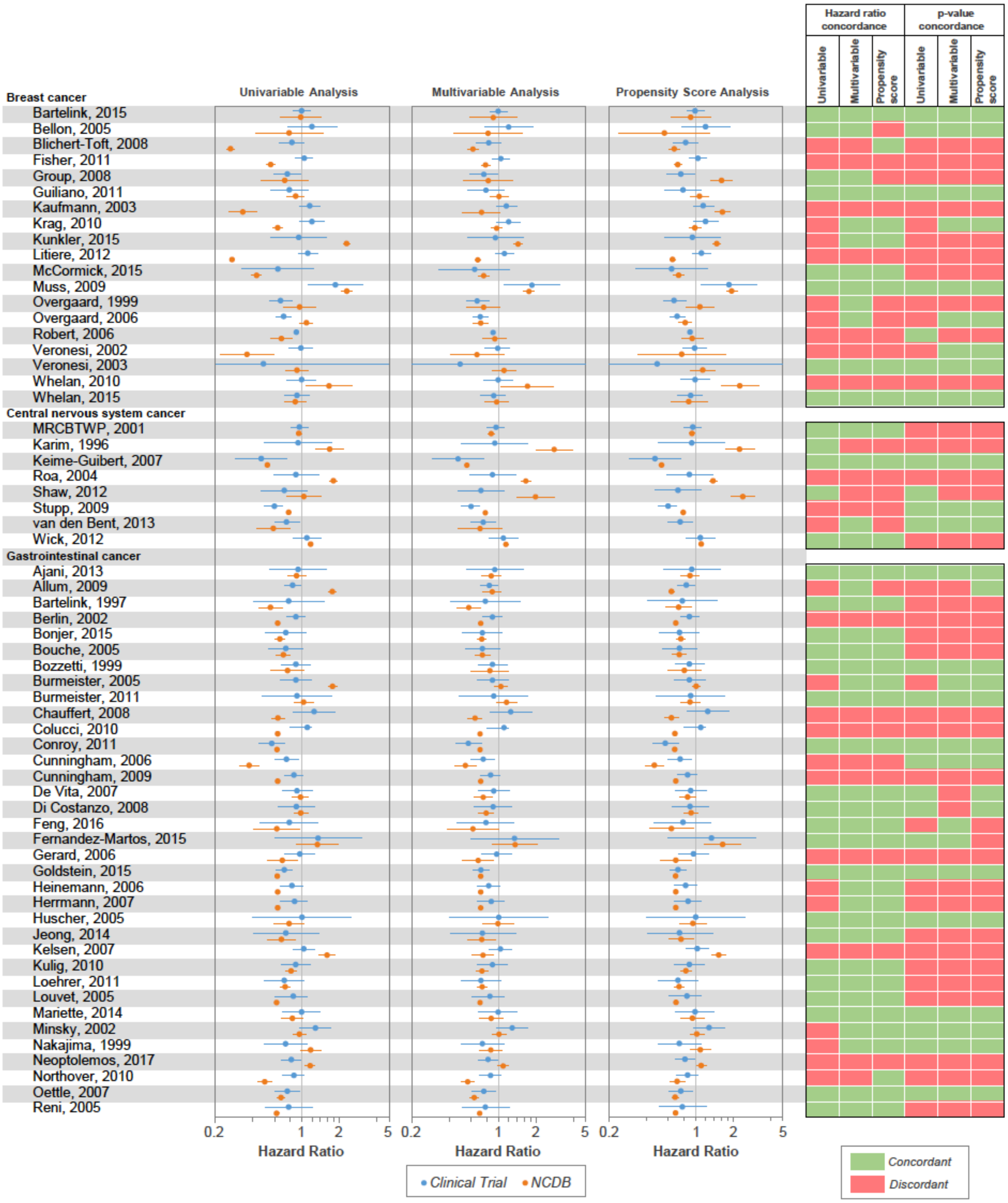

© 2020 Kumar A et al. *JAMA Network Open.*

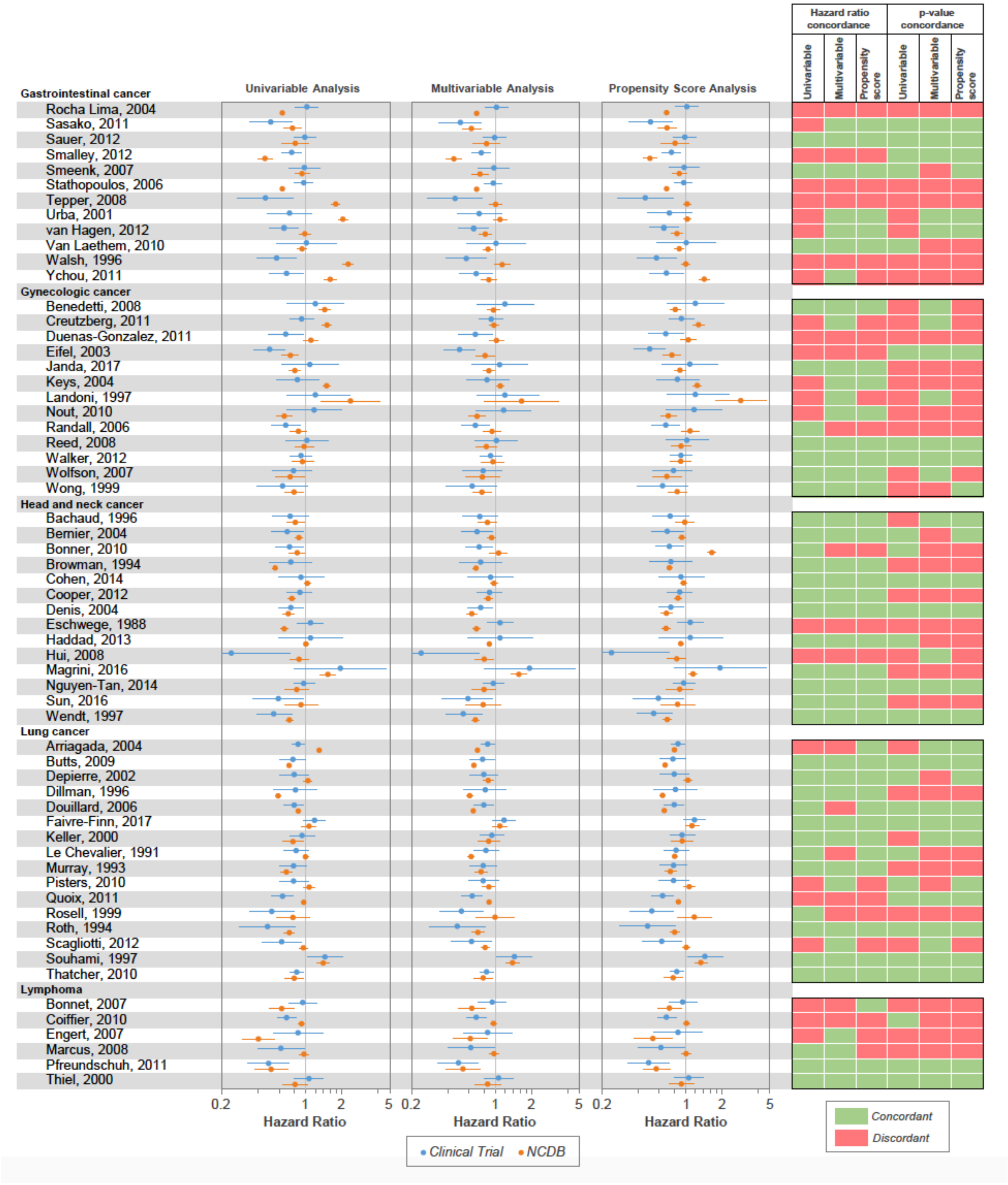

© 2020 Kumar A et al. *JAMA Network Open.*

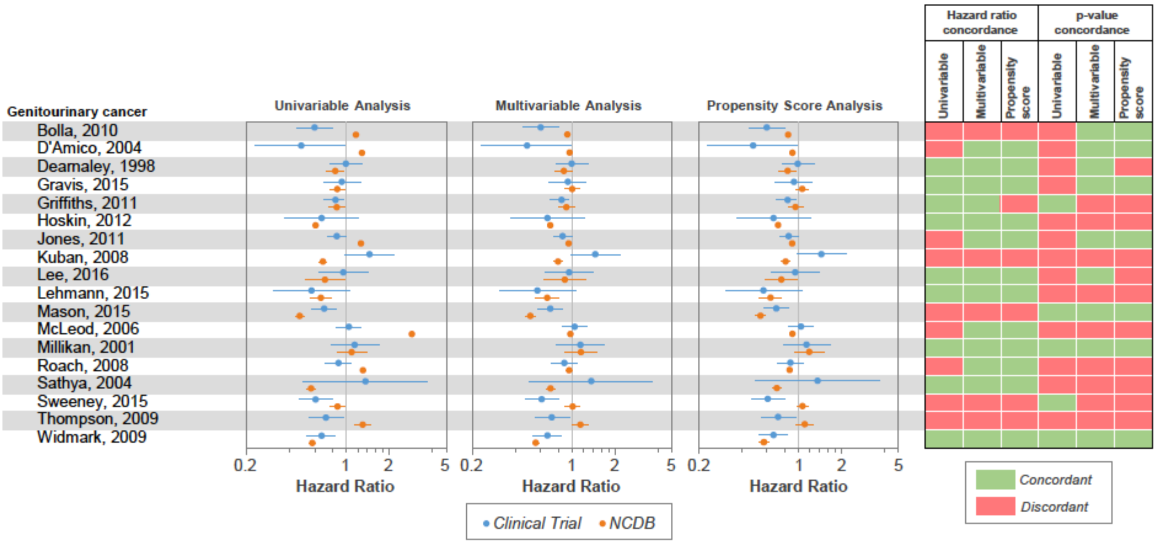

© 2020 Kumar A et al. *JAMA Network Open.*

eTable 1. Examples of clinical trials included and excluded from analysis

**Examples of clinical trial questions included in analysis**	**Examples of clinical trial questions excluded in analysis**
Total Laparoscopic Hysterectomy versus Total Abdominal Hysterectomy on Survival among Women With Stage I Endometrial Cancer	Perioperative chemotherapy with fluorouracil plus leucovorin, oxaliplatin, and docetaxel versus fluorouracil or capecitabine plus cisplatin and epirubicin for locally advanced, resectable gastric adenocarcinoma
Induction chemotherapy versus no induction chemotherapy followed by preoperative chemoradiation in patients with esophageal cancer	FOLFIRINOX versus gemcitabine/Nab-paclitaxel for neoadjuvant treatment of resectable pancreatic head adenocarcinoma
Laparoscopic versus open surgery for rectal cancer	MVAC versus gemcitabine/cisplatin in patients with inoperable metastatic or relapsed urothelial carcinoma
Concurrent chemoradiotherapy versus radiotherapy alone in head and neck cancer	Bendamustine plus rituximab versus R-CHOP or R-CVP in patients with indolent Non-Hodgkin Lymphoma or Mantle-Cell Lymphoma
Observation versus adjuvant chemoradiation in pancreatic cancer	Alectinib versus Crizotinib in ALK-positive Non-Small-Call Lung Cancer

© 2020 Kumar A et al. *JAMA Network Open.*

eTable 2. Concordance in statistical significance between clinical trials and NCDB analysis stratified by whether the NCDB analysis favors more aggressive treatment arm

		Number of trials (%)		
		**Univariable analysis**	**Multivariable analysis**	**Propensity score analysis**
**NCDB analysis significantly favors more aggressive* treatment arm (p<0.05)**		**65 (53)**	**70 (57)**	**68 (55)**
	Clinical trial p<0.05 with clinical trial hazard ratio in the same direction as the NCDB analysis (i.e. agreement in effect direction)	26 (40)	28 (40)	28 (41)
	Clinical trial p<0.05 with clinical trial hazard ratio in the opposite direction as the NCDB analysis (i.e. disagreement in effect direction)	0 (0)	0 (0)	0 (0)
	Clinical trial p≥0.05	39 (60)	42 (60)	40 (59)
**NCDB analysis significantly favors less aggressive* treatment arm (p<0.05)**		**23 (19)**	**7 (6)**	**13 (11)**
	Clinical trial p<0.05 with clinical trial hazard ratio in the same direction as the NCDB analysis (i.e. agreement in effect direction)	0 (0)	0 (0)	0 (0)
	Clinical trial p<0.05 with clinical trial hazard ratio in the opposite direction as the NCDB analysis (i.e. disagreement in effect direction)	10 (43)	1 (14)	4 (31)
	Clinical trial p≥0.05	13 (57)	6 (86)	9 (69)
**NCDB analysis non-significant (p≥0.05)**		**35 (28)**	**46 (37)**	**42 (34)**
	Clinical trial p≥0.05	25 (71)	29 (63)	28 (67)
	Clinical trial p<0.05	10 (29)	17 (37)	14 (33)

Abbreviation: NCDB = National Cancer Database.
* Aggressive treatment arms were defined as those comparing surgery to non-surgical approaches (surgery more aggressive), comparing open surgery to laparoscopic surgery (open surgery more aggressive), comparing chemotherapy to no chemotherapy (chemotherapy more aggressive), or comparing radiation to no radiation (radiation more aggressive). These criteria corresponded to 123 of the 141 trials. Eighteen trials omitted from this subset analysis related to treatments arms lacking a clear aggressive arm (e.g. lumpectomy plus radiation versus mastectomy, pre-operative versus post-operative chemoradiotherapy, whole abdomen radiation treatment versus chemotherapy, etc.)

© 2020 Kumar A et al. *JAMA Network Open.*

eFigure 2. Comparison of hazard ratios from randomized clinical trials and analyses with data from NCDB. Each point on the scatter plot represents the hazard ratio for overall survival from one of the 141 randomized clinical trials in this study and the corresponding analysis within NCDB. Red dots represent NCDB analyses with p-values and findings of the analyses that agreed with the clinical trials (concordant), whereas grey dots represent p-values and findings that disagreed with the clinical trials (not concordant). The grey dashed line represents the line where clinical trial hazard ratios equal NCDB hazard ratios. The individual panels represent univariable analyses (A), multivariable analyses (B), and propensity score analyses (C). *Abbreviations*: NCDB = National Cancer Database.

A

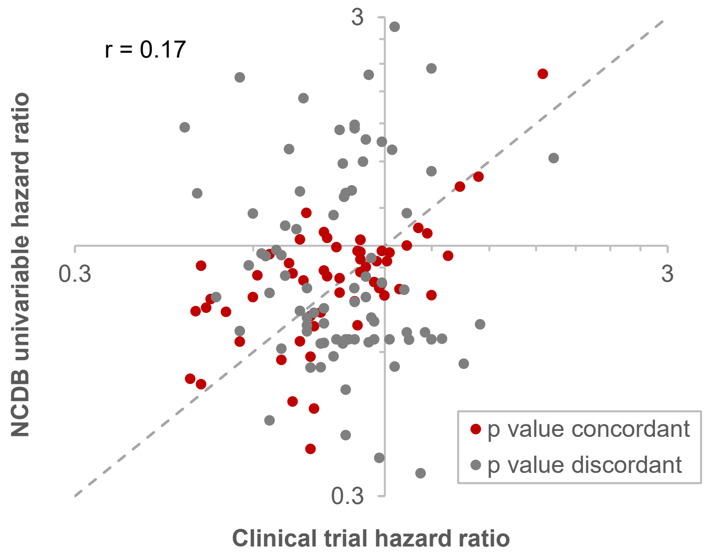

B

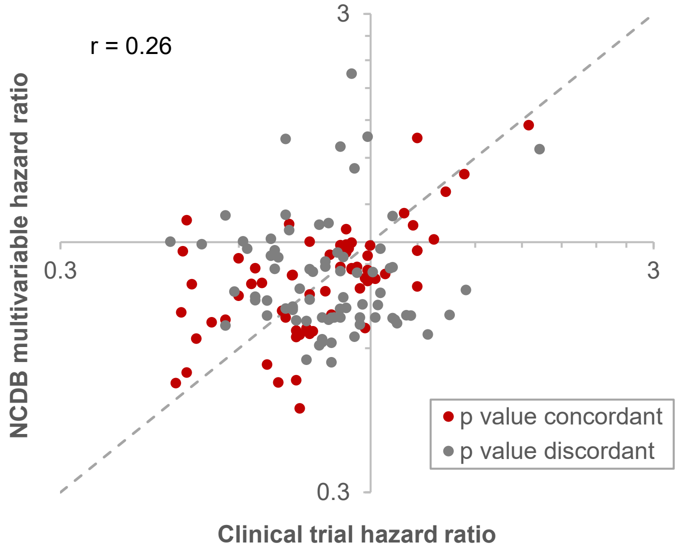

© 2020 Kumar A et al. *JAMA Network Open.*

C

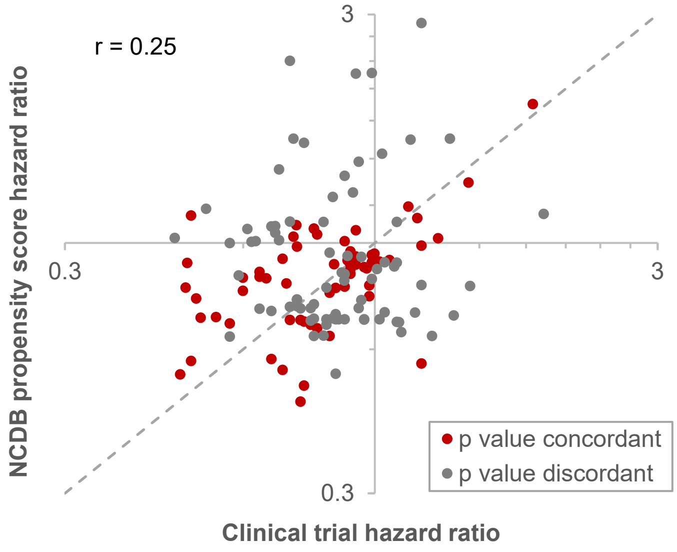

© 2020 Kumar A et al. *JAMA Network Open.*
